# Detection of tissue origin of a 43 kDa diabetogenic protein from alloxan-induced diabetic rats

**DOI:** 10.4103/0973-3930.50711

**Published:** 2009

**Authors:** Shivkumar D. Chauhan, Nirmalendu M. Nath, Vinay K. Tule

**Affiliations:** 1 Eugeniks, Genetic Research Institute, 106/A-wing, First floor, Lokmat Bhavan, Ramdaspeth, Nagpur-440 012, (MS), India; 2 Department of Biochemistry, RTM Nagpur University, LIT Premises, Amravati Road, Nagpur-440 010, (MS), India

**Keywords:** Alloxan diabetes, diabetogenic factor, ionic transporters, oxidant/antioxidant status, polymorphonuclear neutrophils

## Abstract

**BACKGROUND:**

Earlier, we had found high levels of circulating immune complexes (CICs) in the serum of type 2 diabetes mellitus patients along with a novel 43 kDa protein.

**METHODS:**

Different tissues of alloxan-induced, diabetic, male albino rats (200–250 g in body weight) were collected for the present study. Tissue proteins were isolated and separated by 10% SDS-polyacrylamide gel electrophoresis (SDS-PAGE). A primary cell culture of polymorphonuclear neutrophils (PMNs) was used to evaluate the effects of the diabetogenic protein. Cell proliferative index, oxidant/antioxidant status, and ion-transporting ability were chosen as study parameters.

**RESULTS:**

SDS-PAGE of different tissues shows that the diabetic liver alone was the only tissue that contained the 43 kDa protein band compared to the normal liver. *In vitro* effects of the new liver protein on PMNs include significantly decreased cell proliferative activity, increased free radical levels, and decreased levels of antioxidant enzymes as well as ionic transporters. The new liver protein also exhibited protease activity when compared with standard trypsin.

**CONCLUSIONS:**

This study concluded that a novel 43 kDa protein obtained from the livers of alloxan-induced diabetic rats shows protease activity as well as antiproliferative activity. Also, this protein may act as a diabetogenic factor as it elicited a significantly gross elevation in the oxidant status level as well as in the levels of lysosomal enzymes and a decrease in the levels of antioxidative enzymes and ionic transporters of PMNs.

## Introduction

Enhanced concentrations of circulating immune complexes[1–5] and the presence of novel 43 kDa circulating immune complexes (CICs)-associated autoantigen in the serum of type 2 diabetes patients has been well documented in our previous work.[6] It was also noted that this unique protein possessed antigenicity and diabetogenic properties.[6] Although reports of the presence of autoantigens in diabetes have been reported earlier,[7–9] evaluations of these intrinsic antigens for diabetogenicity were never carried out. Furthermore, the tissue origin of such autoantigens remains unexplored probably due to unavailability of human tissues for such studies. Hence, an alternative animal model was developed for the study of the diabetic state.

In the present study, an attempt has been made to examine the presence of an unknown protein in different tissues of alloxan-induced diabetic rats by isolating proteins and separating them by SDS-PAGE. This investigation describes the presence of a new protein moiety in experimentally induced diabetic tissue(s). This protein was evaluated for its antigenicity, the oxidant/antioxidant status, active ionic transport, and lysosomal degranulation after incubation with human neutrophils.

## Materials and Methods

### Experimental animals

Twenty-one male, albino Wistar rats, 18–22 weeks of age, weighing between 200-250 g were used for the study. The animals were maintained in a well-ventilated room with natural day-night cycles in large polypropylene cages. They were fed a balanced rodent pellet diet (Hindustan Liver Pvt. Ltd; Bombay, India) and water *ad libitum* throughout the experimental period. The animals were quarantined for one week prior to the experiments to acclimate them to laboratory conditions. The study protocol was approved by the IAEC (Institutional Animal Ethics Committee, Govt. of India) (Registration No. 414/01/ab/CPCSEA).

### Induction of diabetes

The rats were starved overnight and diabetes was induced by a single subcutaneous injection of alloxan monohydrate (Sigma Chemical Company, St. Louie, USA) (80 mg/kg body weight) dissolved in freshly prepared 0.15 M sodium acetate buffer, pH 4.5.[10] After seven days, the animals were stabilized and considered as diabetic if their blood glucose level was > 200 mg/dL after a single subcutaneous injection of alloxan. Hyperglycemia and glycosuria were evaluated by using a glucoxidase test strip for blood and Benedict's test for urine in relation to normal controls.

### Isolation of protein from tissue

Diabetic rats were sacrificed and tissues from liver, brain, lung, kidney, spleen, pancreas, heart, and muscle were isolated, blotted free from blood, washed with 0.1 M phosphate-buffered saline (PBS), cut into small pieces, and incubated at 60°C for 30 minutes with lysis buffer (containing 100 mM NaCl, 10 mM Tris-HCl, 25 mM EDTA, and 0.5% SDS, pH 7.5). The whole tissue lysates were then centrifuged at 3000 rpm for 20 minutes and 200 μg of the supernatants were then run on 10% SDS-PAGE gels. The gels was developed with silver nitrate reagent or Coomassie Brilliant Blue R-250 stain. The unknown protein band was segregated from the gel by employing an electro-elution technique and tested for antigenicity and/or diabetogenicity.

### Establishment of primary cell culture of PMNs

Normal human polymorphonuclear neutrophils (PMNs) were isolated by using the method of Boyum *et al.*[11] PMNs were cultured in tissue culture plates containing RPMI-1640 medium supplemented with 13% fetal calf serum, penicillin (10,000 IU/mL), streptomycin (100 μg/mL), gentamycin (2 μL/mL), and nystatin (40 μg/mL) and incubated at 37°C in a 5% CO_2_ atmosphere.

### Diabetogenic effect of the hepatic 43 kDa protein

Cultured cells were separated with 0.01% Collagenase-P and approximately 4.2–4.3 × 10^6^ cells were incubated with 100 μM of hepatic protein for 60 min at 37°C. After incubation, the cells were washed twice with 0.1 M PBS and different biochemical parameters were assayed in order to ascertain antigenicity of the 43 kDa hepatic protein from alloxan-induced diabetic rats. Known diabetic human PMNs were isolated and served as a positive control.

### Effect of the hepatic 43 kDa protein on different biochemical parameters

The activity of nitric oxide and superoxide radicals, Cu-Zn superoxide dismutase (Cu-Zn SOD), Na^+^/K^+^-ATPase, Ca^++^/Mg^++^-ATPase, acid phosphatase, and cathepsin D were estimated by the method of Green *et al.,*[12] Johnston *et al.,*[13] Misra *et al.,*[14] Post *et al.,*[15] Lynch *et al.,*[16] Shinowara *et al.,*[17] and Sapalsky *et al.*[18] respectively while the inorganic phosphate level was estimated by the method of Fiske *et al*.[19]

### Evaluation of protease activity of the 43 kDa hepatic protein

Casein (0.5%) was used as a substrate to determine protease activity of the 43 kDa protein. Fifty micrograms of the hepatic 43 kDa protein obtained from alloxan-induced diabetic rats was incubated with 2 mL of 0.5% casein solution at 37°C for 60 minutes. The reaction was stopped by adding 2 mL 30% TCA solution and the reaction mixture was centrifuged at 10,000 rpm for 20 minutes. The concentration of small peptides in the supernatant was estimated by using the method of Lowry *et al.*[20]. Trypsin was used as a positive control (50 μg). Activity is expressed in units and specific activity is expressed as units/mg of protein.

### Effect of the 43 kDa hepatic protein on PMN cell growth

One hundred micrograms of the 43 kDa hepatic protein was incubated with 4.3 × 10^6^ PMNs at 37°C for 60 minutes. After incubation, the cells were washed with 0.1 M PBS and incubated for another 72 h; cell numbers were counted after every six hours.

### Statistical analysis

The data were analyzed using GraphPad Prism software (version 4). The results are expressed as mean ± SD and Student's ‘t’-test was used to assess statistical significance. *P* values < 0.05 were considered to be significant.

## Results

**Figure 1:**
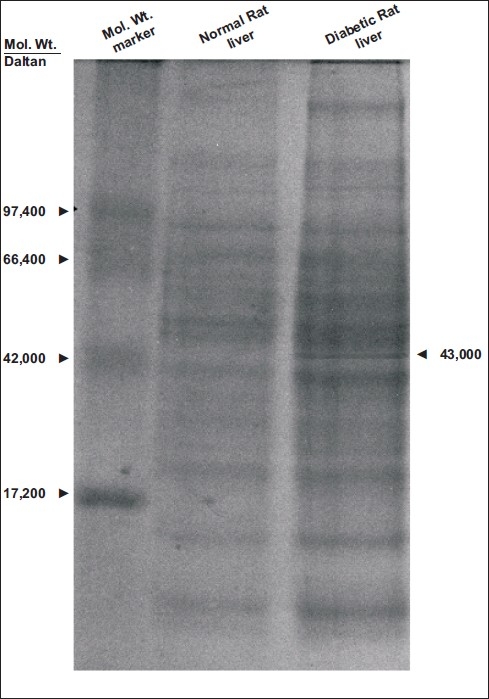
SDS-PAGE electrophoresis pattern of normal and alloxan-induced diabetic liver proteins

**Figure 2:**
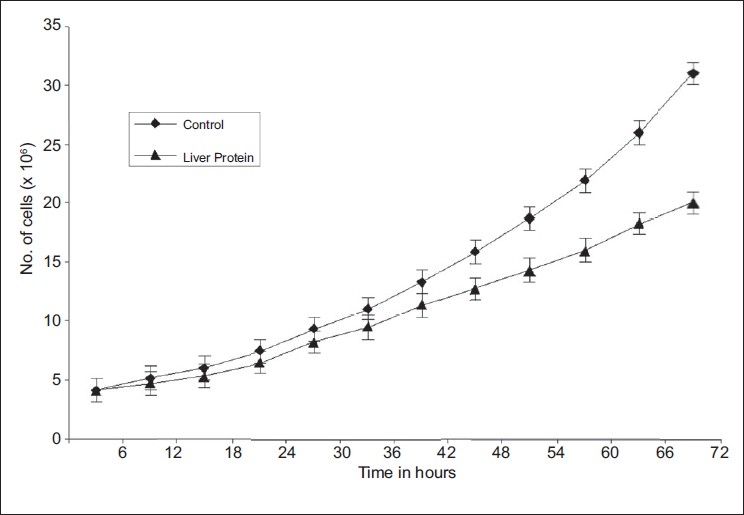
Effect of hepatic 43 kDa protein obtained from alloxan-induced diabetic rats on PMN growth rate; results expressed as mean ± SD of 7 experiments

Examination of the proteins separated by SDS-PAGE of different tissues obtained from alloxan-induced diabetic rats revealed that the diabetic liver alone contained an unidentified protein band when compared the livers from normal (not treated with alloxan) rats. A molecular weight marker indicated that the unidentified protein had a molecular weight of 43 kDa [Figure 1]. The eluate obtained after electro-elution of the above protein from the gel was found to exhibit protease activity it had a significant specific activity (374 ± 12 Units) compared to that of control trypsin (419 ± 9 Units). A significant decrease was noted in the cell growth (*P* < 0.001) when PMN cells were treated with the test 43 kDa liver protein [Figure 2].

Simultaneously, several biochemical aberrations were found in the PMNs after *in vitro* treatment with the protein [Table 1]. The oxidant status of the PMNs was found to be significantly enhanced as the levels of free radicals such as nitric oxide and superoxide radicals were greatly elevated whereas the levels of the antioxidant enzyme (Cu-Zn SOD) were found to be significantly decreased. Concurrent incapacitation of ionic pumps was also observed. The activity of both Na^+^/K^+^ ATPase and Ca^++^/Mg^++^ ATPase was grossly decreased. The evidence of lysosomal degranulation of PMNs enhanced by such treatment has been depicted in Table 1. Table 2 represents the comparative values of all the above biochemical parameters in type 2 diabetic patients' PMNs as compared to normal healthy PMNs. A similar pattern of biochemical changes emerged in pathological (diabetic) PMNs as seen after the *in vitro* incubation of human PMNs with the 43 kDa hepatic protein isolated from the livers of alloxan-induced diabetic rats.

**Table 1:** *In vitro* effect of 43 kDa liver protein obtained from alloxan-induced diabetic rats on the oxidant / antioxidant status, active ionic transporters, and lysosomal enzymes on normal PMNs

Parameters	Control (Without treatment)	Experimental (With treatment)
Nitric oxide radical (pM of nitric oxide released / mg protein)	4.13 ± 0.29	7.06 ± 0.34*
Superoxide radical (nM of cytochrome C reduced / mg protein)	3.81 ± 0.22	6.51 ± 0.33*
Cu-Zn SOD (Units of activity / mg protein)	3.79 ± 0.28	1.24 ± 0.09*
Na^+^/K^+^ ATPase (μM of Pi (inorganic phosphate) librated / min / mg protein)	9.3 ± 0.35	4.8 ± 0.24*
Ca^++^/Mg^++^ ATPase (μM of Pi librated / min / mg protein)	5.7 ± 0.19	2.3 ± 0.11*
Acid phosphatase (μM of Pi librated / min / mg protein)	0.85 ± 0.08	3.04 ± 0.08*
Cathepsin D (nM of tyrosine released / min / mg protein)	16.42 ± 0.61	41.17 ± 1.41*

Results are expressed as mean ± SD of 13 experiments,

* *P* < 0.001 when compared with control

**Table 2:** Levels of oxidant / antioxidant status, ion transporters, and lysosomal degranulation of PMNs from normal as well as diabetic patients

Parameters	Normal	Diabetic
Nitric oxide radical (pM of nitric oxide released / mg protein)	4.18 ± 0.27	7.16 ± 0.31*
Superoxide radical (nM of cytochrome C reduced / mg protein)	3.81 ± 0.22	6.75 ± 0.30*
Cu-Zn SOD (Units of activity / mg protein)	3.70 ± 0.28	1.20 ± 0.10*
Na^+^/K^+^ ATPase (μM of Pi librated / min / mg protein)	9.40 ± 0.35	4.88 ± 0.24*
Ca^++^/Mg^++^ ATPase (μM of Pi librated / min / mg protein)	5.72 ± 0.19	2.3 ± 0.10*
Acid phosphatase (μM of Pi librated / min / mg protein)	0.85 ± 0.08	2.14 ± 0.09*
Cathepsin D (nM of tyrosine released / min / mg protein)	16.42 ± 0.61	42.15 ± 1.36*

Results are expressed as mean ± SD of 13 experiments,

* *P* < 0.001 when compared with normal control.

## Discussion

The existence of various autoantigens in diabetes has been reported by other authors.[7–9,21–26] Earlier,[6] we reported a novel 43 kDa CIC-associated autoantigen in the sera obtained from type 2 diabetes patients. Investigation of the tissue origin of this autoantigen has remained unexplored mainly because of the absence of a suitable model. In this communication, we present the evaluation of this unidentified protein moiety obtained from the livers of alloxan-induced diabetic rats by SDS-PAGE. This study clearly reveals that with the exception of the liver, all tissues including brain, lung, kidney, muscle, heart, spleen, and pancreas exhibited symmetrical protein patterns when compared with normal controls. However, the diabetic liver contained an unidentified protein band which was conspicuously absent in other diabetic tissues. Interestingly, the molecular weight of this new protein was found to be 43 kDa, quite similar to our earlier observation of a similar molecular weight, CIC-associated protein obtained from the serum of type 2 diabetes patients.[6] Although a direct match of these two observations cannot be done to investigate the origin of the novel protein because of the different models employed in the two studies, one cannot totally rule out the possibility of the liver being the originator of this protein in diabetic patients.

Autoantigens are known to be associated with a strong protease activity. The isolated 43 kDa protein obtained from alloxan-induced diabetic livers of rats also exhibited a strong protease activity, suggesting its autoantigenic character. The antiproliferative activity exhibited by this protein on human PMN cell culture is also consistent with the reported antiproliferative action of other autoantigens.[27–28] The CIC-associated, 43 kDa protein obtained from human type 2 diabetic sera had also demonstrated the same characteristics.[6]

*In vitro* incubation of this liver protein with a human PMN primary culture revealed that there was a higher accumulation of free radicals such as superoxide and nitric oxide radicals, significant lowering in the activity of free radical-quenching enzyme (Cu-Zn SOD), gross degranulation of lysosomal enzymes, and a gross decrease in the ionic transport. These changes were consistent with the changes exhibited by the CIC-associated protein from human type 2 diabetic sera documented in our earlier work.[6]

Overall, the striking resemblance in molecular weight, autoantigenic property, and biochemical actions of both the isolated proteins from these two different diabetic sources (type 2 diabetes patient sera and alloxan-induced diabetic rat livers) point towards a plausible connection between them.

## Conclusions

It can be concluded from our findings that a novel 43 kDa protein isolated from the livers of alloxan-induced diabetic rats demonstrated the same protease activity and antiproliferative activity as the 43 kDa protein obtained from CIC of type 2 diabetes patients. Furthermore, this protein acts as a diabetogenic agent as it elicited a gross elevation in the levels of oxidant status as well as lysosomal enzymes and a decrease in the levels of antioxidative enzymes and ionic pumps in PMNs. Thus, the liver may be the key organ where diabetogenic protein(s) is/are generated.
